# Parainfluenza Virus 5 Priming Followed by SIV/HIV Virus-Like-Particle Boosting Induces Potent and Durable Immune Responses in Nonhuman Primates

**DOI:** 10.3389/fimmu.2021.623996

**Published:** 2021-02-25

**Authors:** Peng Xiao, Krista Dienger-Stambaugh, Xuemin Chen, Huiling Wei, Shannon Phan, Ashley C. Beavis, Karnail Singh, Nihar R. Deb Adhikary, Pooja Tiwari, Francois Villinger, Biao He, Paul Spearman

**Affiliations:** ^1^ New Iberia Research Center, University of Louisiana at Lafayette, New Iberia, LA, United States; ^2^ Division of Infectious Diseases, Department of Pediatrics, Cincinnati Children’s Hospital Medical Center and University of Cincinnati, Cincinnati, OH, United States; ^3^ Division of Infectious Diseases, Emory University, Atlanta, GA, United States; ^4^ Department of Infectious Diseases, University of Georgia, Athens, GA, United States; ^5^ Wallace H Coulter Department of Bioengineering, Georgia Institute of Technology, Atlanta, GA, United States

**Keywords:** parainfluenza virus 5 (PIV5), human immunodeficiency virus (HIV) vaccine, virus-like particles (VLP), rhesus macaques, immune responses

## Abstract

The search for a preventive vaccine against HIV infection remains an ongoing challenge, indicating the need for novel approaches. Parainfluenza virus 5 (PIV5) is a paramyxovirus replicating in the upper airways that is not associated with any animal or human pathology. In animal models, PIV5-vectored vaccines have shown protection against influenza, RSV, and other human pathogens. Here, we generated PIV5 vaccines expressing HIV envelope (Env) and SIV Gag and administered them intranasally to macaques, followed by boosting with virus-like particles (VLPs) containing trimeric HIV Env. Moreover, we compared the immune responses generated by PIV5-SHIV prime/VLPs boost regimen in naïve vs a control group in which pre-existing immunity to the PIV5 vector was established. We demonstrate for the first time that intranasal administration of PIV5-based HIV vaccines is safe, well-tolerated and immunogenic, and that boosting with adjuvanted trimeric Env VLPs enhances humoral and cellular immune responses. The PIV5 prime/VLPs boost regimen induced robust and durable systemic and mucosal Env-specific antibody titers with functional activities including ADCC and neutralization. This regimen also induced highly polyfunctional antigen-specific T cell responses. Importantly, we show that diminished responses due to PIV5 pre-existing immunity can be overcome in part with VLP protein boosts. Overall, these results establish that PIV5-based HIV vaccine candidates are promising and warrant further investigation including moving on to primate challenge studies.

## Introduction

Combination antiretroviral therapy (ART) has markedly changed the clinical outlook for human immunodeficiency virus (HIV)-infected patients and represents a major advance in the fight against HIV/AIDS. Viral suppression from ART has been shown to reduce sexual transmission of HIV, leading to the concept of treatment as prevention as a proven intervention to slow the spread of the epidemic (1–3). Despite this advance, there still are an estimated 1.7 million new HIV infections per year worldwide (4). The development of a safe and effective HIV vaccine remains the most promising strategy for preventing infections on a global basis and eventually ending the HIV pandemic (5–7). Phase III clinical trials of HIV vaccines evaluated thus far have not convincingly led to protection from infection. The RV144 trial employing a recombinant canarypox (ALVAC-HIV) prime and envelope (Env) gp120 protein (AIDSVAX B/E) boost vaccine approach showed evidence of a modest protective effect, and gave hope that a protective HIV vaccine is ultimately achievable (8, 9). However, the more recent failure of HVTN 702 in South Africa, which utilized the same poxvirus prime/gp120 boost strategy, underlines the need for novel approaches (10). The immune correlates required for an HIV vaccine to provide protection in human populations remain incompletely defined. In the absence of well-established correlates of protection, desirable characteristics of an HIV vaccine include the induction of robust and long-lasting functional antibodies against Env and antigen-specific T cell polyfunctional responses for controlling viral replication in the event of infection (11, 12). Vaccine-elicited mucosal immune responses against HIV are less well explored but are also desirable characteristics of vaccine candidates (13).

Parainfluenza virus 5 (PIV5) is a single-stranded, negative-sense RNA virus of the genus Rubulavirus in the family Paramyxoviridae (14). It has several distinguishing characteristics that make it an attractive vector for vaccine development. PIV5 can infect a variety of animals but causes no known disease, and is also non-pathogenic in humans (15–18). Its general use as a kennel cough live vaccine in dogs underscores its safety. PIV5 can be produced in high titers in many cell lines, including Vero cells, which have been approved for use for vaccine production by FDA (19). Furthermore, PIV5 has been used to develop vaccine candidates that elicit mucosal and systemic immune responses and are protective in preclinical studies against diverse viral and bacterial pathogens, including influenza virus, rabies virus, respiratory syncytial virus, and Mycobacterium tuberculosis (20–24).

HIV-1 Env proteins rarely result in the elicitation of highly functional antibodies, even when delivered in the presence of adjuvants (25, 26). Neutralizing antibodies against primary (Tier 2) isolates have been particularly hard to generate in human trials, even when Env-specific antibody titers are robust (27, 28). SOSIP Env trimers have recently shown promise in inducing neutralizing antibodies against homologous Tier 2 HIV isolates in nonhuman primates (29–32). SIV virus-like particles (VLPs) have demonstrated the ability to enhance the titer and breadth of antibody responses in nonhuman primates when employed as a boost following live vector priming (33). Here we employed VLPs displaying native trimeric HIV Env on an SIV Gag core (SHIV VLPs) as a booster following PIV5 priming. We sought to determine if boosting with VLPs enhanced the magnitude and quality of antibody and T cell responses primed by a PIV5-based HIV vaccine regimen.

The efficacy of live biologic vector-based vaccines, such as recombinant viral or bacterial vectors, can be limited by pre-existing immunity against vectors from natural exposure or vaccine-induced immunity (34–37). Therefore, one critical question for the use of PIV5 as a vector, is whether prior exposure to PIV5 would limit the induction of immune responses by PIV5-based vaccines. A previous study has shown that pre-existing neutralizing antibodies against PIV5 in dogs did not negatively affect the immunogenicity of a PIV5-based influenza vaccine (38). However, this potential issue remains to be addressed in nonhuman primates or in humans.

In this study, we found that an HIV vaccine regimen employing an intranasal (IN) prime with recombinant PIV5 vector followed by boosting with SHIV VLPs elicited robust and durable systemic responses, including polyfunctional T cell responses and antibodies mediating neutralization and ADCC. Pre-existing immunity to PIV5 resulted in lower responses to the prime, but these differences could be partly overcome following the VLPs boost.

## Materials and Methods

### Construction of Recombinant Parainfluenza Virus 5-Based Vaccine

Madin-Darby bovine kidney (MDBK) cells were cultured in Dulbecco’s modified Eagle medium (DMEM) with 5% fetal bovine serum (FBS), 5% l-glutamine, 100 IU/ml penicillin, and 100 μg/ml streptomycin (Sigma-Aldrich). The recombinant PIV5 vectors expressing HIV-1 JRFL Env (gp140), ΔSH-SIVmac239 Gag (SivGag p55), or Ebola virus glycoprotein (EboG) were generated as described before (22). Briefly, plasmids containing full-length genome of PIV5 with the gp140 gene between SH and HN of PIV5, SivGag lacking SH coding sequence and insertion of EboG between HN and L were constructed following standard molecular cloning techniques. Infectious viruses were rescued using these plasmids along with necessary support plasmids encoding PIV5 replication machinery. The rescued viruses were plaque-purified and confirmed by RT-PCR sequencing. The viruses were propagated in MDBK cells as previously described (22). Recombinant PIV5 viruses were harvested at 7 days post infection, clarified by centrifugation at 3,000 rpm for 10 min, aliquoted and stored at -80°C. PIV5 virus titers were determined by plaque assay as previously described (24). MDBK cells were infected with each recombinant PIV5-based HIV vaccine (at an MOI of 5) or were infected with PIV5-mock. At 24 or 48 h post infection, cell lysates were separated by SDS-PAGE, transferred to PVDF membranes, and blotted with a monoclonal antibody specific to the HIV-1 Env (D7324) or SIV Gag (CA183), respectively.

### Generation and Characterization of SHIV Virus-Like Particles

SHIV VLPs were generated from stable, inducible cell lines expressing the full-length, codon-optimized HIV-1 Env (JRFL strain) and codon-optimized SIV Gag (SIVmac239 strain). pcDNA5/TO-puro was constructed from backbone pCDNA5/TO (ThermoFisher Scientific) as previously described (39). Codon-optimized Env and Gag genes were synthesized by a commercial supplier (GenScript) and were cloned into pcDNA5/TO-puro (Env) or pcDNA4/TO (ThermoFisher Scientific) (SIV Gag) using EcoR1 and Xba1 cloning sites. 293F/6TR cells (39) were cultured in FreeStyle 293 Expression medium (ThermoFisher Scientific) supplemented with 5 µg/ml blasticidin (Invivogen). The 293F cells were transfected with pCDNA4/TO SIVGag and pCDNA5/TO-puro HIV Env plasmids using Lipofectamine 2000 (ThermoFisher Scientific). 48 h later, cells were transferred into fresh media supplemented with 10 µg/ml zeocin and 1 μg/ml puromycin (Invivogen) and antibiotic-resistant cells were selected. Stable cells were induced with 2 µg/ml doxycycline for 48 h, and cleared supernatants containing HIV Env and SIV Gag VLPs (referred to as SHIV VLPs) were harvested and fractionated on 20%–60% sucrose gradients by ultracentrifugation. Fractions were analyzed by Western blotting for the presence of HIV Env and SIV Gag using specific antibodies as described above. The refractive index of each fraction was measured, and their buoyant density calculated. Ebola GP VLPs were generated as described previously (39). To prepare large-scale VLPs batches, stable VLPs-producing 293F/6TR cells were grown in 1L Ehrlenmeyer flasks and induced with doxycycline for 48 to 72 h. Supernatants were harvested, filtered through 0.45-μm-pore-size filters, and purified and concentrated by pelleting through 20% sucrose. VLPs were resuspended in PBS and quantified for Env content.

### Animals

Ten healthy male adult Indian rhesus macaques (Macaca mulatta) were used in this study. Six macaques were included in group 1 (body weight, averages 10.06 ± 0.22 kg; age, averages 9.93 ± 0.07 years). Four macaques were included in group 2 (body weight, averages 10.01 ± 0.35 kg; age, averages 10.00 ± 0.01 years). Two MamuA*01+ macaques were evenly distributed in two groups. Animals were housed and maintained at the New Iberia Research Center (NIRC) of the University of Louisiana at Lafayette in accordance with the rules and regulations of the Committee on the Care and Use of Laboratory Animal Resources. The entire study (protocol 8778-075) was reviewed and approved by the University of Louisiana at Lafayette Institutional Animal Care and Use Committee (IACUC). All animals were negative for SIV, simian T cell leukemia virus and simian retrovirus.

### Study Design and Immunization

**Figure 1:**
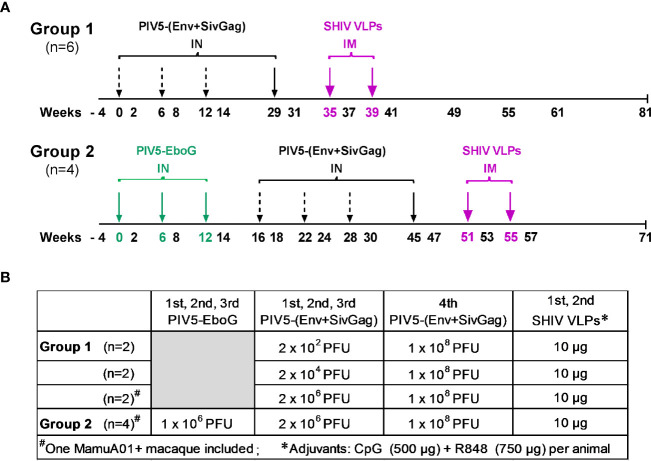
Schematic diagram of immunization regimen and dose administrations. **(A)** Animals in Group 1 were sequentially primed intranasally (IN) at weeks 0, 6, 12, and 29 with PIV5-based SHIV vaccine, followed by adjuvanted SHIV VLPs boosts intramuscularly (IM) at weeks 35 and 39, respectively. Animals in Group 2 were preimmunized with PIV5-EboG vaccine intranasally at weeks 0, 6, and 12 prior to initiating the same prime/boost immunization regimen in Group 1 at week 16. **(B)** Vaccination immunogens and administration doses are summarized for each study group.

The study consisted of two experimental groups. Group 1 was a dose-ranging study evaluating PIV5 prime/VLP boost immunization regimen, with the primary endpoint of defining the immunogenicity and optimal dosing of this regimen. Group 2 received a similar PIV5 prime/VLP boost regimen, but was pre-immunized with PIV5 bearing a heterologous insert of EBOV GP, in order to investigate whether pre-existing immunity would hamper the induction of SHIV specific humoral and/or T cell immune responses. The immunization regimen and doses are detailed in **Figure 1**. Six rhesus macaques in group 1 were administrated PIV5-(Env+SivGag) intranasally (IN) at weeks 0, 6, and 12 with three different doses: 2 × 10^2^ PFU (n=2), 2 × 10^4^ PFU (n=2) and 2 × 10^6^ PFU (n=2), respectively, and all received the same 4^th^ dose (1 × 10^8^ PFU, IN) at week 29, followed by two intramuscular (IM) HIV VLPs boosts (35 and 39 weeks). VLP boosts consisted of purified VLPs (containing 10 μg gp120 Env) premixed with CpG (500 µg) and R848 (750 µg) adjuvants (40). The four animals in group 2 were all pre-immunized with PIV5-EboG vaccine (1 × 10^6^ PFU, IN) at weeks 0, 6, and 12 to induce pre-existing immunity to PIV5 prior to initiating the same prime/boost immunization regimen, as outlined in **Figure 1**.

### Sample Collection

Peripheral blood mononuclear cells (PBMCs) obtained throughout the immunization course were Ficoll-separated and used fresh for ELISPOT assay or T-cell intracellular cytokine staining (ICS) assay. Plasma samples were collected, aliquoted, and stored at -80°C until analyzed. Bronchoalveolar lavage (BAL) fluid samples were collected following each immunization and bone marrow (BM) samples were collected at the end of study. Nasal and rectal secretions were collected by using Merocel sponges and processed as previously described (41), and stored at -80°C until analyzed.

### ELISPOT Assay

Total and antigen-specific plasmablasts in peripheral blood or plasma cell responses in bone marrow were determined by ELISPOT assay as previously described (42). Briefly, 96-well multiscreen HTS filter plates (Millipore) were coated overnight at 4°C with 100 μl/well of 5 μg/ml of goat anti-monkey IgG or IgA antibodies (Rockland) or of 5 μg/ml of HIV gp120 (Immune Tech) or Ebola GP protein (IBT Bioservices), respectively. Plates were washed with PBS-0.05% Tween 20 (PBS-T) and blocked with complete medium at 37°C for 2 h. Freshly isolated cells were plated in duplicates in serial 3-fold dilutions and incubated overnight in a 5% CO_2_ incubator at 37°C. Plates were washed with PBS-T and incubated with biotin-conjugated anti-monkey IgG or IgA antibodies (Rockland) diluted 1:1,000 for 1 h at 37°C. After washing, plates were incubated with horseradish peroxidase (HRP)-conjugated streptavidin diluted 1:1,000 (Vector labs) at room temperature and developed using the AEC substrate kit (BD Biosciences). To stop the reaction, plates were washed extensively with water followed by air drying. Spots were imaged and counted using the Immunospot ELISPOT Analyzer (Cellular Technology Limited). The number of spots specific for each Ig isotype was reported as the number of either total or antigen-specific antibody producing cells per million.

### Envelope Protein Enzyme-Linked Immunosorbent Assay

Systemic and mucosal binding antibodies to HIV Env protein were assessed by enzyme-linked immunosorbent assay (ELISA) as described previously (39, 43). The antibody titer was defined as the reciprocal of the average test sample dilution at which the optical density (OD) of the test sample (run in triplicate) was greater than the preimmunized control sample (run in triplicate) plus three standard deviations, at the same dilution.

### SIV Gag Protein Quantitative Western Blot Analysis

SIV Gag antibody responses were assessed by quantitative Western blot analysis. 100 ng (Env-equivalent) of SHIV VLPs were loaded into a single lane and separated on a 4%–12% Bis-Tris NuPAGE gel (Invitrogen), then transferred to nitrocellulose (Invitrogen). Blots were rinsed with water and placed inside the multi-channel MPX blotting system (LI-COR Biosciences). Total SIV Gag protein was detected using Revert 700 Total Protein Stain Kit (LI-COR Biosciences). Each lane of the MPX device was stained for 5 min then rinsed twice with kit wash buffer, imaged and quantitated in the 700 channel on an Odyssey CLx Infrared Imaging System (LI-COR Biosciences). Blots were replaced in the same orientation in the MPX device and blocked with Odyssey blocking buffer (LI-COR Biosciences) for 1 h before addition of diluted serum (1 to 50 in Odyssey blocking buffer + 0.1% Tween20) and incubated overnight at 4°C. Channels were washed five times with wash buffer (1X PBS + 0.05% Tween-20) before blots were removed from MPX device and probed with Goat anti-Human-IgG IRDye 800CW. The SIV Gag-reactive IR800 signal was imaged and quantitated (background subtracted) and total SIV Gag signal (background subtracted) was used to normalize load. Data are reported as fold change over normalized pre-immune plasma sample signal.

### TZM-bl Neutralization Assay

The HIV-1 primary cell-free viruses were obtained from the NIH AIDS Reagent Program and used in TZM-bl neutralization assay adapted from Dr. Montefiori’s laboratory at Duke University. All viruses were propagated in human peripheral blood mononuclear cells activated with Concanavalin A and recombinant interleukin-2 (IL-2). Virus stocks were titrated in TZM-bl cells to derive the 50% tissue culture infective dose (TCID_50_). For neutralization assay, viruses were titrated at a concentration that resulted in 10^5^ relative light units (RLU). Briefly, 50 µl of 2-fold serial dilutions of heat-inactivated plasma and 50 µl of titrated virus were mixed and incubated for 1 h at 37˚C in a 96-well flat-bottom plate. Next, 100 µl of TZM-bl cells (10,000/well) in complete DMEM medium containing 15 µg/ml diethylaminoethyl (DEAE) dextran (Sigma-Aldrich) were added to each well, and the 96-well plates were incubated for 48 h. Assay controls included TZM-bl cells alone (cell control, no virus) and TZM-bl cells with virus only (virus control, no test sample). At 48 h, the cells were lysed and luciferase activity was measured immediately on a BioTek luminometer. Neutralization activities were determined by GraphPad Prism (v7.01) software, where values from the experimental wells were compared with the value from control wells. The 50% inhibitory concentration (IC50) was calculated based on the plasma dilution that caused a 50% reduction of RLU compared to the control wells.

### Antibody Dependent Cellular Cytotoxicity Assay

ADCC was measured using HIV-infected targets and was performed as previously described (44). Briefly, target cells of CEM.NKR.CCR5.CD4+-Luc were infected with 50 ng of HIV-1 JRFL virus and cultured for 4 days. Two-fold serial dilutions of each heat-inactivated plasma sample were added to the infected targets for 20 min at room temperature. CD16-KHYG-1 effector cells were added at a 10:1 effector-to-target ratio and incubated for additional 8 h. The cells were lysed and luciferase activity was measured immediately on a BioTek luminometer. Fifty percent (50%) ADCC killing indicates the plasma titers required for half-maximal cell lysis (similar to 50% inhibitory concentration [IC50] for neutralization).

### Neutralizing Antibody Titers to Parainfluenza Virus 5

PIV5 neutralizing antibody titers were measured by ELISA as described previously (38). Briefly serial dilutions of monkey plasma were incubated with 200 TCID_50_ of PIV5 virus and incubated for 2 h before being added to 96-well plates containing 90% confluent MDBK cells. After 3 days of incubation, the wells were scored after staining with mouse anti PIV5 followed by FITC labeled goat anti-mouse Ig. The neutralizing antibody titer was the highest plasma dilution completely neutralizing 200 TCID_50_ of PIV5.

### T Cell Intracellular Cytokine Staining Assay

To detect cytokine production by T cells, freshly isolated PBMC (2×10^6^) at two weeks post each immunization were stimulated with peptide pools of HIV Env or SIV Gag at a 1-μg/ml final concentration in the presence of anti-CD28 ECD (1 μg/ml, clone CD28.2; Beckman Coulter), anti-CD107a FITC (1 μg/ml, clone eBioH4A3; eBioscience) and anti-CD49d (1 μg/ml, clone 9F10; BD Biosciences). Cells were cultured for 2 h at 37°C before adding Brefeldin A (10 μg/ml, BD Biosciences) for an additional 4 h. For each assay, an unstimulated control (dimethyl sulfoxide, DMSO only) and a positive control (PMA/Ionomycin) were included. After 6 h, cells were washed with PBS and an Aqua viability dye (Invitrogen) was used to stain for Live/Dead cells at room temperature (RT) for 15 min. Cells were washed with PBS containing 2% fetal bovine serum (FBS), and surface stained for 30 min at 4°C with the following antibodies: anti-CD3 Alexa700 (clone SP34-2; BD Biosciences), anti-CD4 BV605 (clone OKT4; BioLegend), anti-CD8 BV450 (clone RPA-T8, BD Biosciences), and anti-CD95 PE-Cy5 (clone DX2, BD Biosciences). Cells were washed with 2% PBS, fixed with Cytofix/Cytoperm (BD Biosciences), permeabilized with 1× Perm/Wash, and incubated with anti-gamma interferon (IFN-γ) PE-Cy7 (clone B27; BD Biosciences), anti-tumor necrosis factor alpha (TNF-α) APC-Cy7 (clone Mab11; BioLegend), anti-interleukin 17A (IL-17A) PE (clone eBio64CAP17; eBioscience) and anti-macrophage inflammatory protein 1 beta (MIP-1β) APC (clone FL34Z3L; eBioscience) antibodies. After 30 min of incubation at 4°C in the dark, cells were washed once with 1× Perm/Wash and once with 2% PBS and resuspended in 2% formaldehyde solution for acquisition on a BD FACSAria Fusion cell sorter. CD3+ T cells were gated for CD4+ and CD8+ T cells, and each population was further phenotyped into memory/naïve subsets by gating CD28 and CD95. The net percent of cytokine-secreting cells was determined by subtraction of the values obtained with DMSO only stimulated samples. Boolean combinations and cytokine positive cells frequency were determined with FlowJo software (version 9.2; ThreeStar Inc). Polyfunctional responses of CD4+ and CD8+ T cells were assessed using the Pestle 2.0 and SPICE 6.0 software (https://niaid.github.io/spice/; Vaccine Research Center, NIAID, NIH) (45).

### Statistical Analyses

Differences between the two groups were evaluated by the exact Wilcoxon rank sum test. The one-way ANOVA test was used for comparisons across three or more groups. The Spearman rank correlation test was used to assess the relationships between two-group parameters. The distribution of the polyfunctional responses was analyzed by using Student’s t-test and permutation comparison test for pie charts between groups. All statistical analyses were considered significant if they produced P values of < 0.05.

## Results

### Generation and Characterization of Parainfluenza Virus 5 and Virus-Like Particles Vaccines

**Figure 2:**
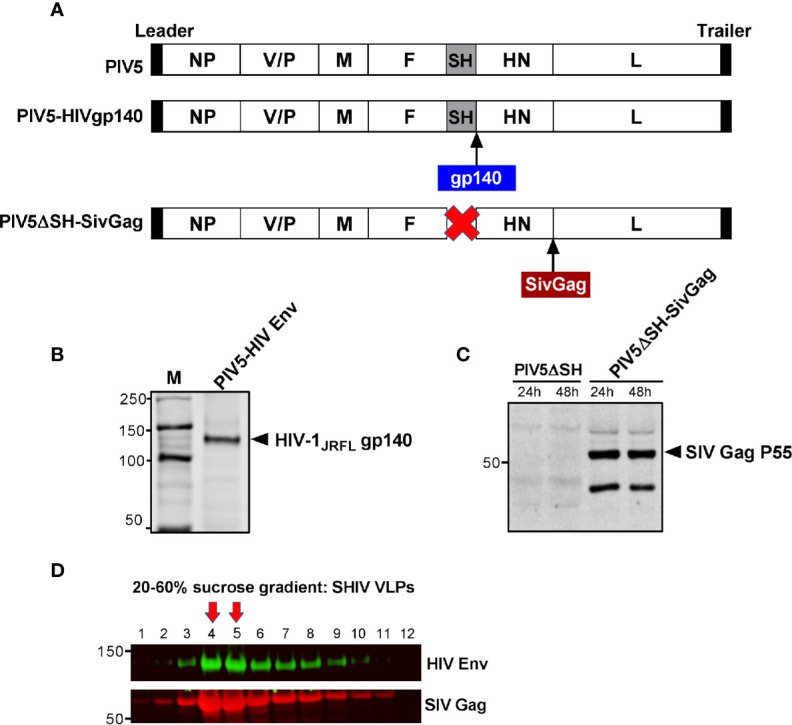
Generation and characterization of PIV5 and VLPs vaccines. **(A)** Schematics of PIV5- HIVgp140 and PIV5ΔSH-SivGag vaccine constructs. NP, nucleoprotein; V, V protein; P, phosphoprotein; M, matrix protein; F, fusion protein; SH, small hydrophobic protein; HN, hemagglutinin-neuraminidase protein; L, RNA-dependent RNA polymerase. Western blot analysis of PIV5 expressing **(B)** HIV-1 JRFL gp140, and **(C)** SIVmac239 Pr55Gag. **(D)** SHIV virus-like particles (VLPs) produced in 293F cells were analyzed by fractions collected from a 20% to 60% sucrose gradient using monoclonal antibody specific to HIV gp120 (top) or SIV Gag (bottom). Red arrows indicate the fractions with highest amount of Env and Gag in VLPs.

Previously, we reported that the SH-HN junction of PIV5 was the optimal site for inserting the hemagglutinin of influenza virus to give the best protection against infection (24). We also found that deletion of the SH gene resulted in a more efficacious vector (46). To generate PIV5-based HIV vaccine candidates, we inserted HIV-1 JRFL gp140 at the SH-HN junction to generate PIV5-HIVgp140 and inserted SIV Gag between HN and L in a PIV5 vector that included a deletion of the SH gene to produce PIV5ΔSH-SIVGag (**Figure 2A**). To test for expression and packaging of recombinant PIV5-based HIV vaccines, MDBK cells were infected with recombinant PIV5 viruses or were mock infected. Supernatants were collected and analyzed by SDS-PAGE and Western blot. Protein bands at sizes appropriate for HIV gp140 protein (**Figure 2B**) and SIV Pr55^Gag^ (**Figure 2C**) from PIV5 expressing vectors were readily visible in test samples. Similarly, we successfully generated a PIV5 expressing a heterologous Ebola glycoprotein from the Ebolavirus Zaire (EBOV) strain, termed PIV5-EboG for the study (data not shown).

Next, we generated SHIV VLPs as booster immunogen using an inducible 293F cell expressing SIVmac239 Gag and HIV-1 JRFL Env. An uncleaved Gag core was included in the VLP design, as we have previously shown that this creates an immunogen with stable Env incorporation under a variety of conditions (47). Electron microscopic analysis of SIV Gag-Env particles budding from the plasma membrane was previously shown (33). To further confirm that SHIV VLPs were generated, cellular supernatants were harvested and centrifuged on a 20 to 60% sucrose gradient, followed by Western blotting. As expected, fractions 14 and 15 corresponding to lanes 4 and 5, respectively (density of 1.16 g/ml) were highly enriched in VLPs, as measured by Env and Gag in these fractions (**Figure 2D**). Purified VLPs used for immunization were quantified by gp120 ELISA.

### Evaluation of Antigen-Specific Plasmablast Responses in Blood and Long-Lived Plasma Cell Responses in Bone Marrow

**Figure 3:**
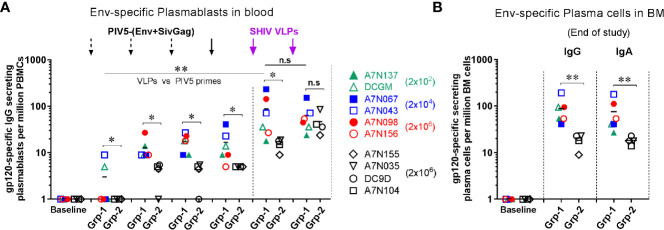
Induction of antigen-specific plasmablast responses in peripheral blood and long-lived plasma cells in bone marrow from both groups. Comparison of Env-specific **(A)** plasmablast responses at day 5 after each immunization, and **(B)** plasma cells responses in BM at the end of study between Group 1 (Grp-1, Week 81) and Group 2 (Grp-2, Week 71). One-way ANOVA test was used to compare the 1st VLPs boost to the all PIV5 primes differences among 5 groups. Three black dashed arrows mark PIV5-(Env+SivGag) primes with different doses (IN) and one black solid arrow marks the fourth prime. Purple solid arrows mark the SHIV VLPs boosts (IM). The level of significance is indicated by P-values as follows: *P < 0.05; **P < 0.01. n.s, *P > *0.05, not significant.

Plasmablasts and plasma cells are short- and long-lived antibody secreting cells (ASC), respectively. To assess the ASC response to the vaccine regimen we initially examined the dynamics of antigen-specific plasmablasts in the peripheral blood over the course of immunization. We found that Env-specific IgG plasmablasts were induced steadily following each of the 4 PIV5-(Env+SivGag) prime immunizations in group 1, but were boosted significantly upon the 1^st^ SHIV VLPs boost (*P*<0.01) (**Figure 3A**). Of note, there was no consistent dose response noted among the animals receiving different doses of PIV5 SHIV vaccine for the first three doses. PIV5 pre-immunized Group 2 animals exhibited a significantly lower level of Env-specific IgG plasmablast responses after immunization with the four PIV5-SHIV vaccines and the 1^st^ VLPs booster compared to animals in group 1 (*P*<0.05). However, the HIV Env plasmablast levels obtained after the 2^nd^ SHIV VLPs boost were equivalent to those seen in Group 1 animals (**Figure 3A**). A similar pattern was also observed for Env-specific IgA producing plasmablasts in the two groups (data not shown).

We also evaluated the frequencies of long-lived plasma cells (LLPCs) in the bone marrow (BM) that are responsible for the steady state antibody production. Env-specific IgG and IgA plasma cells in BM were detected at the end of study for group 1 (week 81, 42 weeks post-2^nd^ SHIV VLPs boost) and group 2 (week 71, 16 weeks post-2^nd^ SHIV VLPs boost). Animals in group 1 had relatively high levels of Env-specific IgG and IgA LLPCs at the end of study, which were significantly higher than those measured in group 2 animals (*P*<0.01) (**Figure 3B**), suggesting attenuation of memory humoral responses by PIV5 pre-existing immunity.

### Evaluation of Systemic and Mucosal Antibody Responses

**Figure 4:**
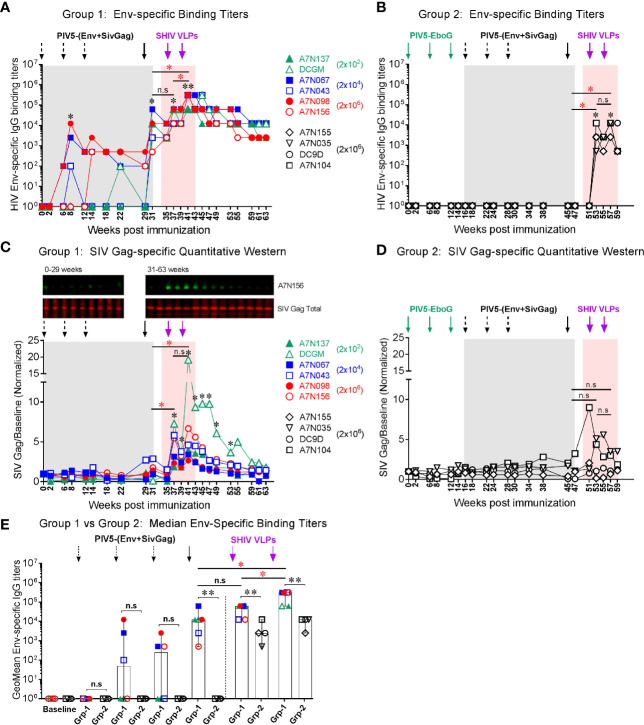
Vaccine-induced systemic binding antibody responses. Env-specific IgG binding titers were determined at the indicated time points in **(A)** Group 1, and **(B)** Group 2, respectively. **(C)** SIV Gag responses for Group 1 animals measured by quantitative Western blotting. A representative blot is shown at the top (time points from one animal). Quantitation was performed on the LiCor Odyssey instrument for all animals and plotted versus baseline. **(D)** SIV Gag responses in Group 2 animals, platted as in **(C)**. **(E)** Comparison of median Env-specific binding titers between Group 1 (Grp-1) and Group 2 (Grp-2) at the indicated time points receiving the same immunization, respectively. The asterisk marks the level of binding IgG titer was significantly induced after indicated immunization compared to the baseline (*P < 0.05; **P < 0.01). The statistical differences were alos examined between the last prime and first and 2nd VLPs boost and illustrated the significant p value as red asterisks. Three black dashed arrows mark PIV5-(Env+SivGag) primes with different doses (IN) and one black solid arrow marks the fourth prime. Purple solid arrows mark the SHIV VLPs boosts (IM). Each dot represents an individual animal in each group. The level of significance is indicated by P-values as follows: *P < 0.05; **P < 0.01. n.s, *P > *0.05, not significant.

To evaluate systemic humoral immune responses, plasma samples were collected at multiple time points after each of the PIV5 prime and the VLPs boost immunizations. In group 1 animals, binding IgG antibody responses to homologous HIV Env gp120 were detectable as early as week 8 (2 weeks post 2^nd^ PIV5 immunization). These titers were markedly boosted at week 31 (2 weeks post 4^th^ PIV5 prime), without any significant difference between the animals primed initially with 2×10^2^, 2x10^4^ or 2x10^6^ PFU PIV5 doses (Fig 4A). Binding antibody responses were significantly enhanced after the 2^nd^ SHIV VLPs boost (week 41, *P*<0.01), with median peak titers of 3.1 ×10^5^ (**Figure 4A**). These responses showed excellent longevity in memory with a median titer of 1.2 ×10^4^ by 10 weeks which were maintained at these levels for the next 20 weeks. In contrast, Group 2 animals showed no detectable seroconversion to HIV Env after four PIV5 SHIV vaccinations, indicating that pre-existing immunity to the vector limited their responses (**Figure 4B**). Only after VLPs boosting were HIV Env titers detectable, with a median peak titer of 1.2 ×10^4^, significantly lower compared to the group 1 (*P*<0.01). The differences between non-immune and vector-immune animals was even more apparent when plotted as the geometric mean of Env specific IgG (**Figure 4E**). This figure emphasizes that Group 2 animals failed to mount an Env-specific response until after the first VLPs administration. Gag-specific immune responses were measured by quantitative Western blotting in Group 1 animals, shown in **Figure 4C**. This method was chosen because of a favorable signal-to-noise ratio as compared to ELISA in our hands. Specific responses were detectable following the 4^th^ immunization with PIV5 vectors, but became much more prominent following the SHIV VLPs boost (**Figure 4C**). Gag responses were near background levels in Group 2 but were significantly enhanced following VLPs boosting (**Figure 4D**). There were no significant correlations between any plasmablast levels measured after the various booster immunizations (PIV5 and VLPs) and corresponding plasma titers for HIV Env antibodies (*P*>0.05).

**Figure 5:**
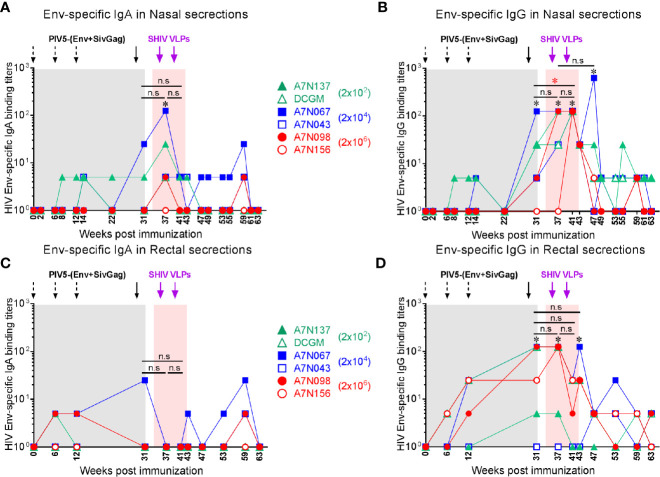
Vaccine-induced mucosal antibody responses in Group 1. **(A)** Env-specific IgA and **(B)** IgG in nasal secretions at the indicated time points. **(C)** Env-specific IgA and **(D)** IgG in rectal secretions at the indicated time points. *, the level of mucosal IgA or IgG was significantly higher after each indicated immunization compared to the baseline (*P* < 0.05). The statistical differences were examined between the last prime and first and 2nd VLPs boost (or week 43 or week 49) and illustrated the significant p value as red asterisks.

Stimulation of antiviral immunity in mucosal immune compartments may be desirable in an HIV vaccine, because HIV transmission occurs primarily at mucosal sites. We therefore investigated whether this intranasal prime/parenteral boost immunization regimen elicited virus-specific mucosal antibody responses. Env-specific IgA and IgG antibodies were detected at low levels in nasal and rectal secretions at week 8 in a few animals, and IgG titers significantly increased at week 31 after the 4^th^ PIV5 prime (*P*<0.05) correlating with the plasma IgG titers. Both nasal IgA and IgG titers were significantly increased at week 37 after the 1^st^ SHIV VLPs boost (*P*<0.05) (**Figures 5A, B**). These responses declined and were maintained at low levels for the next 20 weeks. Env-specific IgA levels in rectal secretions were low and only observed in a few animals even after the 1^st^ SHIV VLPs boost (**Figure 5C**). Of note, Env-specific rectal IgG levels were significantly increased after the 4^th^ PIV5 prime (*P*<0.05) in 4 out of the 6 monkeys and after the 1^st^ and 2^nd^ SHIV VLPs boosts (*P*<0.05) (**Figure 5D**). In contrast to Group 1, mucosal antibodies were completely undetectable in Group 2 after the 4 PIV5 SHIV immunizations, and barely detectable after two SHIV VLPs boosts (data not shown). Overall, despite employing an intranasal prime, the vaccine did not elicit robust mucosal antibody responses. However, there was some evidence of parenteral boosting of PIV5-primed mucosal IgA and IgG responses.

### Antibody Dependent Cellular Cytotoxicity Activity Against Human Immunodeficiency Virus -1

**Figure 6:**
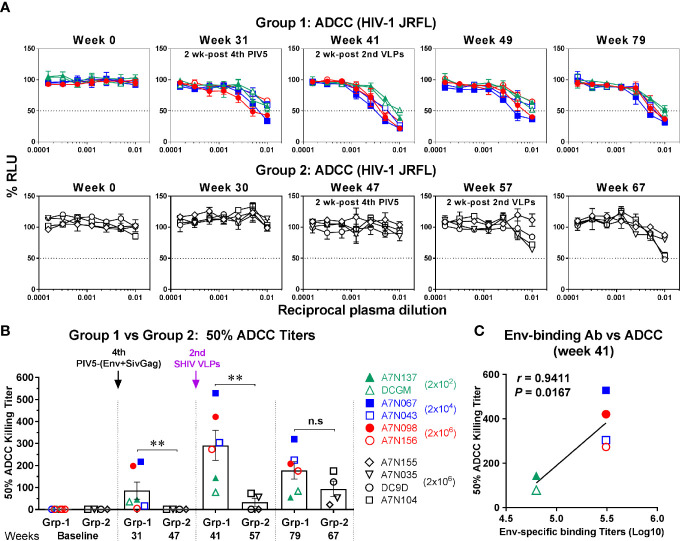
ADCC activity against HIV-1. **(A)** Plasma samples in Group 1 (upper panel) and Group 2 (lower panel) were tested with CD16-KHYG-1 effector cells to kill CEM.NKR.CCR5.CD4+-Luc target cells infected with HIV-1 JRFL. ADCC activity was measured as the dose-dependent loss of luciferase activity in relative light units (RLU). The dotted line indicates half-maximal infection or 50% ADCC killing (cell lysis) titers. **(B)** Comparison of 50% ADCC killing titers between Group 1 (Grp-1) and Group 2 (Grp-2) at the indicated time points receiving the same immunization, respectively (solid arrows). Error bars indicate the standard errors of the means. Each dot represents an individual animal. Statistical analyses were considered significant of P values as follows: * *P <*0.05; ** *P <*0.01. n.s, *P >*0.05, not significant. **(C)** Correlation between Env-specific IgG binding titers and 50% ADCC killing titers in Group 1 (week 41). The correlation coefficients (r) and P values are from Spearman rank analysis.

To determine whether the vaccine-induced binding antibodies were functional, we initially tested them for non-neutralizing ADCC activity against homologous (HIV-1 JRFL), a tier 2 virus. ADCC activity mediated by Env-specific IgG binding antibody was detectable in three animals of Group 1 at week 31 (2 weeks after the 4^th^ PIV5 vaccine), and all demonstrated ADCC at week 41 (2 weeks after the 2^nd^ SHIV VLPs) (**Figure 6A**, upper panel). Although the ADCC titer weakened somewhat by week 49, ADCC activity was still detected at week 79 (**Figure 6A**, upper panel). In contrast, the ADCC titers in Group 2 were essentially negative except for two animals at week 67 (**Figure 6A**, lower panel). A comparison of both groups for corresponding time points relative to PIV5 SHIV prime/VLPs boost immunization for the 50% ADCC titers (half-maximal cell lysis) showed significantly higher ADCC titers in Group 1 compared to the Group 2 (*P*<0.01) (**Figure 6B**), except for the last time point analyzed. There also was a significant correlation between Env-specific IgG binding titer and 50% ADCC titers for Group 1 animals at week 41 (*r*=0.9411, *P*=0.0167) (**Figure 6C**). However, there was no such correlation for Group 2 (data not shown).

### Neutralizing Activity to Human Immunodeficiency Virus-1

**Figure 7:**
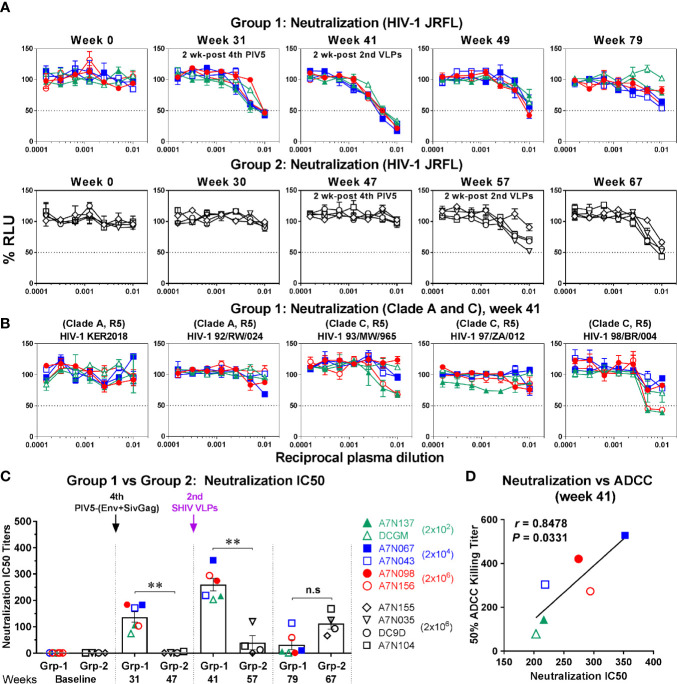
Neutralization against HIV-1. **(A)** Plasma samples in Group 1 (upper panel) and Group 2 (lower panel) were incubated with HIV-1 JRFL and tested for the ability to block viral infectivity (as the dose-dependent loss of luciferase activity in RLU) using the TZM-bl neutralization assay. The dotted line indicates half-maximal infection or 50% neutralization (IC50). **(B)** Neutralization against the indicated HIV-1 viruses (clade A or C, tier 2) was measured in Group 1 (week 41). **(C)** Comparison of neutralization IC50 titers between Group 1 (Grp-1) and Group 2 (Grp-2) at the indicated time points receiving the same immunization, respectively (solid arrows). Error bars indicate the standard errors of the means. Each dot represents an individual animal. Statistical analyses were considered significant of P values as follows: *P < 0.05; **P < 0.01. n.s, *P > *0.05, not significant. **(D)** Correlation between neutralization and ADCC activity against HIV-1 JRFL in Group 1 (week 41). The correlation coefficients (r) and P values were derived from Spearman rank analysis.

Next, we determined whether antibody-neutralizing activity was induced following PIV5 immunization and SHIV VLPs boosts. Virus neutralization was tested against both homologous (HIV-1 JRFL) and heterologous (clade A and C, tier 2) HIV-1 isolates using the TZM-bl assay. Similar to ADCC, Env-specific neutralizing antibodies were detected in Group 1 monkeys at week 31 (2 weeks after the 4^th^ PIV5 vaccine), and the peak neutralizing titers were reached at week 41 (2 weeks after the 2^nd^ SHIV VLPs boost) (**Figure 7A**, upper panel). Unlike ADCC, the neutralizing antibody titers gradually declined over time (**Figure 7A**, upper panel). We further tested Group 1 week 41 plasma samples for neutralization against heterologous HIV-1 viruses (clade A or C). However, among the five heterologous isolates tested, neutralization was observed only against HIV-1 98/BR/004 virus, for two animals (**Figure 7B**). In contrast to Group 1, neutralization titers were only detected in two animals in Group 2 by week 57 (2 weeks post the 2^nd^ SHIV VLPs) and 67 (**Figure 7A**, lower panel). A comparison of the 50% neutralization titers (IC50) between the two groups showed statistical significant differences (*P*<0.01), except for the last time point tested (**Figure 7C**). Moreover, we observed a striking correlation between neutralization and ADCC activity against HIV-1 JRFL in Group 1 monkeys at week 41 (*r*=0.8478, *P*=0.0331) (**Figure 7D**). No such correlation was observed for Group 2 (data not shown).

### Evaluation of Antigen-Specific T Cell Responses

**Figure 8:**
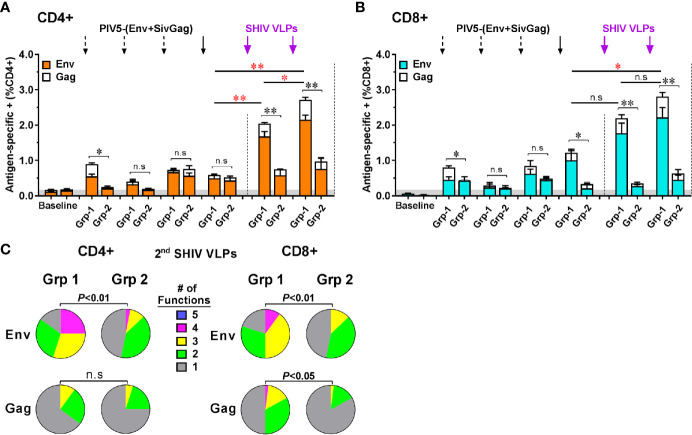
Characterization of vaccine-induced antigen-specific CD4+ and CD8+ memory T cells responses by intracellular cytokine staining for cells secreting IFN-γ, TNF-α, IL-17A, MIP-1β, and CD107a. Stacked responses to HIV Env and SIV Gag peptides pool by **(A)** CD4+ and **(B)** CD8+ cells between Group 1 (Grp-1) and Group 2 (Grp-2). Three black dashed arrows mark PIV5-(Env+SivGag) primes with different doses (IN) and one black solid arrow marks the fourth prime. Purple solid arrows mark the SHIV VLPs boosts (IM). The statistical differences were examined between the last prime and first and 2nd VLPs boost and illustrated the significant p value as red asterisks. **(C)** Env- and Gag-specific CD4+ and CD8+ T cells polyfunctional responses between two groups. Pie charts represent the distribution of cytokine profile with mono-(grey), bi-(green), tri-(yellow), tetra-(pink), or penta-(blue) combinatorial functions. Analyses were performed with the Pestle and SPICE software. Student’s t-test and permutation test were used for pie comparison between two groups. The level of significance is indicated by P-values as follows: *P < 0.05; **P < 0.01. n.s, *P > *0.05, not significant.

We investigated the induction of Env- and Gag-specific memory CD4+ and CD8+ T cell responses in PBMC at 2 weeks post each prime and boost immunization *via* intracellular cytokine staining for T cells secreting of IFN-γ, TNF-α, IL-17A, MIP-1β, and CD107a following HIV Env or SIV Gag peptide pool stimulation (**Figure S1**). We found that both CD4+ and CD8+ T cells specific for the HIV Env and SIV Gag were expanded and detectable in Group 1 monkeys including those administered the lowest (2 × 10^2^ PFU) intranasal dose of PIV5 (**Figure S2**). The sum of the responses by group are illustrated in **Figures 8A, B** for CD4 and CD8 T cell responses respectively with a predominance of responses to HIV Env rather than SIV Gag. These responses were low but detectable after even the first PIV5 immunization in Group 1, but markedly boosted after VLPs boosts (**Figures 8A, B**). In contrast, T cell responses in Group 2 monkeys were significantly lower for both CD4 and CD8 T cells (*P*<0.01). We next evaluated the polyfunctional responses of antigen-specific CD4+ and CD8+ T cells, defined by those cells that simultaneously produce multiple cytokines. As shown in **Figure 8C**, Env-specific CD4+ and CD8+ responses in Group 1 were highly polyfunctional (*P*<0.01) compared to those in Group 2, with more than 50% cytokine secreting cells exhibiting three (yellow) or four (pink) functions, though no combination of five (blue) were detected. In addition, the IFN-γ+ TNF-α+ CD107a+ secreting cells were the most representative T cell population induced in Group 1 monkeys. No statistical difference was observed for Gag-specific polyfunctional CD4+ T cell responses between the two groups. However, highly polyfunctional Gag-specific CD8+ T cells were induced in Group 1 with about 20% of three or four functions compared to that of 2% in Group 2 (*P*<0.05) (**Figure 8C**). Indeed, the Group 2 had a significantly higher percentage of monofunctional cytokine profile compared to the Group 1.

### Parainfluenza Virus 5 Neutralizing Antibody Responses to Successive Immunizations

PIV5 neutralizing antibodies became detectable as early as 2 weeks after the initial immunizations reaching a titer plateau comprised between 10^2^ and 10^3^ by week 8 (**Figure S3**) in animals from both groups, irrespective of the initial doses of PIV5 vaccine in Group 1 (data not shown). These vector-specific neutralizing responses clearly diminished the initial response to immunizations using the same vector in Group 2 (**Figures 3**–**8**), although there was still antigen priming as judged by the binding antibody response to a single VLP boost (**Figure 4B**). Of note also, the PIV5 neutralizing antibody titer plateau suggests that these responses reached a maximum titer following two intranasal doses that was not increased by additional immunizations.

## Discussion

The development of a safe and effective vaccine regimen to prevent HIV infection or disease remains an elusive goal. Evaluating new vectors and prime-boost combinations is warranted as part of the quest for a protective vaccine. PIV5 is a well characterized live, nonpathogenic virus strain that has been shown to elicit protective responses against multiple viral and bacterial pathogens in animal models, but had not previously been evaluated as an HIV vaccine candidate vector. In this study, we evaluated the immunogenicity of IN administration of recombinant PIV5 vectors expressing HIVgp140 and SIV Gag, followed by boosting with SHIV VLPs displaying trimeric Env on their surface as a mucosal and systemic-targeted vaccine candidate regimen. We included SIV Gag and HIV Env in the vector design, a combination chosen with future SHIV challenge experiments in mind to establish protective efficacy. SHIV VLPs were created that matched the antigens included in the live vector, and were administered parenterally with the expectation that magnitude and durability of B and T cell responses would be enhanced.

The study provided three important observations. First, IN immunizations with PIV5-based HIV vaccine elicited potent systemic and more limited mucosal antibody titers, functional antiviral antibodies mediating ADCC and neutralization, and SHIV-specific T cell responses. Second, boosting with TLR7/8/9-adjuvanted VLPs markedly enhanced antiviral responses at the systemic level in both magnitude and functionality, as demonstrated by higher levels of ADCC, neutralizing antibodies, and the development of polyfunctional antigen-specific CD4+ and CD8+ T cell responses. Notably, antibody responses were maintained for a substantial duration following this prime-boost regimen. Third, pre-existing immunity against PIV5 (induced by PIV5 EboG) markedly reduced HIV and SIV-specific immune responses.

Inhibition of mucosal HIV acquisition is thought to rely primarily on neutralizing antibodies based on passive protection experiments in macaque models (48–51). However, there is also accumulating evidence for a role for Fcγ receptor-mediated effector antibody functions to (such as ADCC) contribute to protection (8, 9, 52–54). We therefore evaluated the magnitude and functionality of humoral responses elicited by the PIV5 prime/VLPs boost regimen. Binding antibody levels were generated with intranasal PIV5 priming, and were significantly boosted by a late dose of PIV5 (at week 29) and by VLPs administration (**Figure 4A**). ADCC responses were also generated by PIV5 administration, and were substantially boosted by VLPs administration (**Figure 6**). Remarkably, Env-specific antibodies persisted at high levels 24 weeks following the last VLPs boost, and ADCC responses were also long-lived. Neutralizing antibody responses, in contrast, were greatest 2 weeks following the second VLPs boost and then rapidly waned (**Figure 6A**). We observed a correlation between neutralization and ADCC activity against HIV-1 JRFL at the peak time point (**Figure 7D**), indicating that these two functions may overlap *in vivo* following this vaccine regimen. Overall, the magnitude, functionality, and durability of the humoral responses were encouraging, supporting plans to address protective efficacy in future studies. We note, however, that neutralization was limited to the homologous JRFL virus, indicating that further refinements will be needed to achieve the difficult goal of substantial neutralization breadth.

T-cell polyfunctionality has been considered an important parameter reflecting the quality of a potent T-cell response (55). Such responses have been demonstrated in HIV-1-infected individuals who are able to control virus without ART and inversely correlate with viremia and disease progression (56, 57), suggesting that CD8+ T-cell polyfunctionality would be a correlate of virus control (58, 59). Conversely, lack of T-cell polyfunctionality would lead to cell exhaustion and functional impairment (60, 61). Thus, the capacity of vaccine induced T cells to produce IFN-γ, TNF-α, IL-17A, MIP-1β, and CD107a was evaluated in this study. We found that PIV5/VLPs vaccine generated Env-specific CD4+ and CD8+ T-cells that were polyfunctional, with >50% of cytokine secreting cells exhibiting three or four functions. Polyfunctional responses were less profound in Group 2 animals with pre-existing immunity. A significant proportion of Gag specific CD8+ T-cells was also induced, which could be advantageous for control of acquired virus. In contrast, monofunctional cells dominated the Gag-specific CD8+ T cell responses induced in Group 2 animals.

Group 1 animals received different amounts of the priming vectors for the initial three doses in an attempt to derive the optimal dose of PIV5 in this model. Statistically-significant differences were not found between the animals administered doses ranging from 2 x 10^2^ to 2 x 10^6^ PFU of SHIV PIV5 in Group 1, although there was a trend towards more reliable responses with higher doses. This was especially apparent for ADCC titers, suggesting a potential association between the PIV5 dose and antibody maturation. The antibody maturation process is dependent on both time and amount of antigen exposure, and our data suggest that the lowest (2x10^2^ PFU) dose was suboptimal (**Figure 6**). However, only minimal differences were observed for neutralization antibody titers relative to the different PIV5 doses (**Figure 7**).

Mucosal IgA antibodies have been associated with protection or delayed acquisition of SHIV (62–64). Intranasal immunization induces antigen-specific immune responses in the upper and lower respiratory tract and genital tract (65, 66). It is hypothesized that intranasal PIV5 vaccine immunization leads to the attraction and maturation of ASCs localized to the mucosa. While we detected Env-specific mucosal IgA in both nasal and rectal secretions following immunization, these levels were very low prior to VLPs boosting. Although IgA is the most abundant antibody isotype in mucosal secretions, we noted that vaccine-induced Env-specific IgG antibodies were higher than those of IgA in both nasal and rectal secretions, indicating that mucosal IgG seen here may represent transudate from the systemic compartment.

Group 2 animals demonstrated a marked reduction in cellular and humoral immune responses that was elicited by induction of vector-specific immunity. This effect was surprising to us, and seemed contradicted by the increasing immune response induced by successive PIV5 immunization in Group 1. Indeed, humoral responses were markedly boosted by PIV5-Gag and PIV5-Env immunization at week 29 (**Figures 4**–**7**), as were cell-mediated immune responses (**Figure 8**). The reasons for the discrepancy between the effectiveness of the day 29 dose in Group 1 and the suppression of PIV5-elicited responses by pre-immunizing with PIV5 in Group 2 are not certain, but we can consider several possibilities. First, the timing of the initial SIV Gag/HIV Env PIV5 immunization in Group 2 was 4 weeks after the last EboG PIV5 administration, whereas the fourth SHIV PIV5 immunization in Group 1 animals was given 17 weeks following the prior dose of PIV5. Thus, it is possible that anti-PIV5 responses had diminished by week 29. A second possibility is that HIV- and SIV-specific anamnestic responses were less inhibited by pre-existing antibodies than was the induction of initial responses (priming). This idea is supported by studies showing that boosting a response requires less antigenic stimulation than initiating a *de novo* immune response (67–70). A third possibility stems from our observation that humoral responses elicited by EboG PIV5 surpassed those elicited for SHIV by PIV5 (data not shown) suggesting that the EboG be a more potent immunogen, potentially acting as an adjuvant for PIV5 specific responses. The suppression of responses to the vector insert in Group 2 is different from with the one we had previously shown in dogs pre-immunized with PIV5, where subsequent administration of PIV5 expressing influenza hemagglutinin successfully generated influenza-specific immune responses (38). It is possible that there is a species difference in their responses to PIV5. This finding, coupled with the fact that levels of detectable anti-PIV5 titers in canine owning humans are lower than in dogs, suggest that giving multiple doses of PIV5-vectored vaccines may remain an effective mucosal vaccine strategy for human immunization.

In conclusion, we have shown that a PIV5-based SHIV vaccine has the ability to induce potent systemic humoral and cell-mediated responses, and more limited mucosal antiviral responses. Boosting with adjuvanted trimeric Env VLPs promoted robust and durable systemic antibody responses and polyfunctional T cell responses, with a limited boosting of mucosal responses. While a stringent induction of pre-existing anti PIV5 immunity clearly affected the development of such potent SIV and HIV-specific responses, the significance of this in human populations remains to be determined. Our results support the further preclinical development of PIV5-based vectors as HIV vaccine candidates, including determination of protective efficacy in nonhuman primate models.

## Data Availability Statement

The raw data supporting the conclusions of this article will be made available by the authors, without undue reservation.

## Ethics Statement

The animal study was reviewed and approved by IACUC, University of Louisiana at Lafayette.

## Author Contributions

BH, PS, and FV designed the study. PX, KD-S, XC, HW, SP, KS, NA, and PT performed the analyses. PX, KS, FV, BH, and PS redacted the manuscript. All authors contributed to the article and approved the submitted version.

## Conflict of Interest

The authors declare that the research was conducted in the absence of any commercial or financial relationships that could be construed as a potential conflict of interest.

## Supplementary Material

Supplementary Figure 1
**(A)** Flow cytometry gating strategy used to identify total memory CD4+ and CD8+ T cell responses in PBMC. **(B)** Representative ICS staining of CD8+ cells in Group 1 after the 2^nd^ SHIV VLPs boost. Flow dot plots illustrate individual cytokine with no stimulation, HIV Env (N and C) peptides or with SIV Gag peptides pool stimulation. A positive control stimulation with PMA/Ionomycin was included in each assay.

Supplementary Figure 2 Induction of antigen-specific cytokine producing CD4+ and CD8+ cells by intranasal administration of different PIV5 doses. **(A, B)** Group 1 of CD4+ or CD8+ T cells secreting cytokines specific for the sum of Env and Gag after administration with three different PIV5 doses of 2×10^2^ PFU (green), 2×10^4^ PFU (blue) and 2×10^6^ PFU (red), respectively. **(C, D)** Group 2 of CD4+ or CD8+ T cells secreting cytokines specific for the sum of Env and Gag after administration with a single PIV5 dose of 2×10^6^ PFU (grey or earth yellow).

Supplementary Figure 3
**(A)** Comparison of titers of anti-PIV5 neutralizing antibodies between Group 1 and Group 2 at the indicated time points receiving the same immunization, respectively. **(B)** Correlation of anti-PIV5 titers relative to PIV5 doses at weeks 2, 8 and 14. The correlation coefficients (r) and P values were derived from Spearman rank analysis. Significant P values are in red font.
