# Supplementary figures and images for: Parainfluenza Virus 5 Priming Followed by SIV/HIV Virus-Like-Particle Boosting Induces Potent and Durable Immune Responses in Nonhuman Primates
Source: Front Immunol. 2021 Feb 25;12:623996. doi: 10.3389/fimmu.2021.623996 (PMC7946978; doi:10.3389/fimmu.2021.623996)

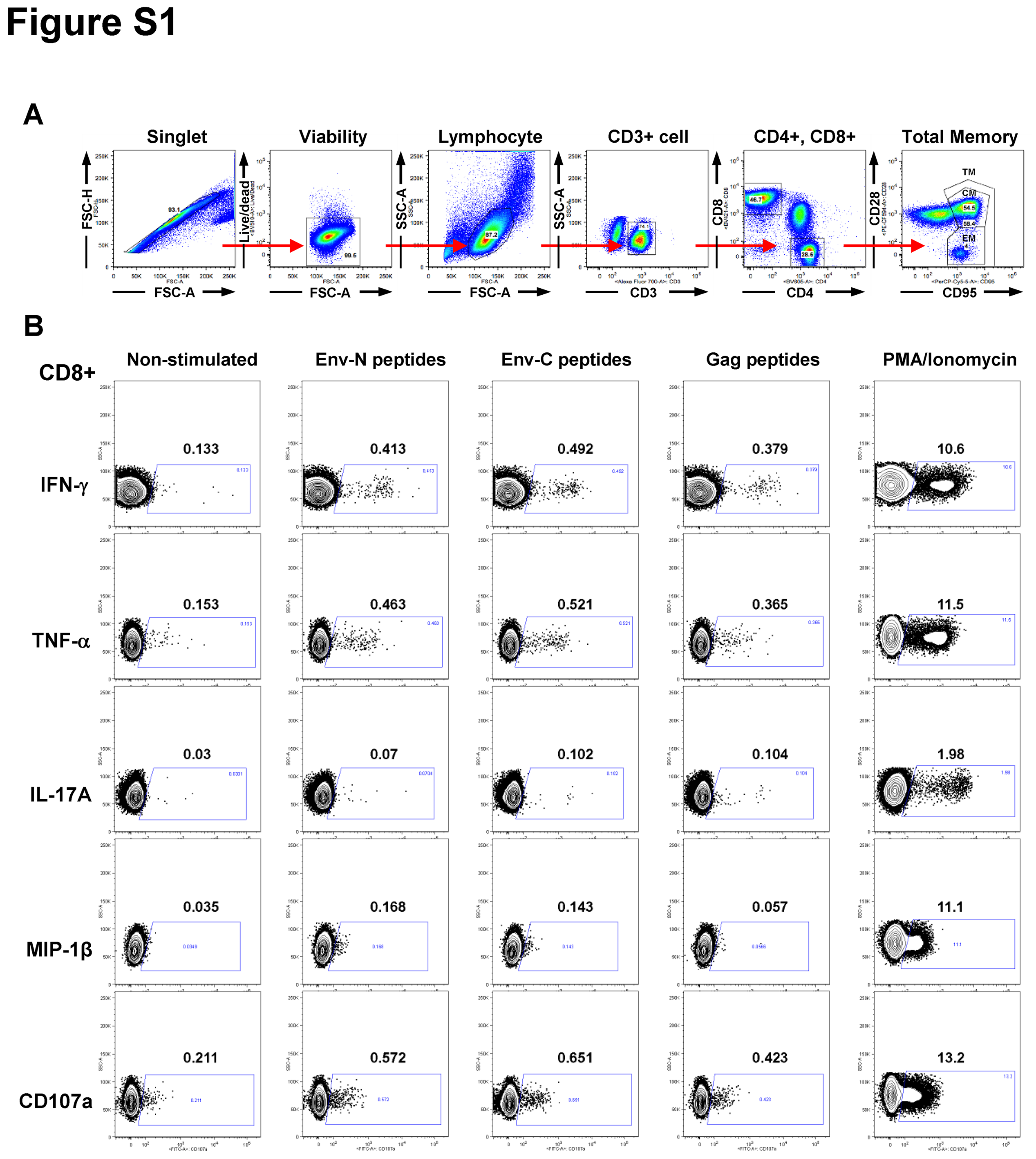

Supplement: Supplementary Figure 1 — (A) Flow cytometry gating strategy used to identify total memory CD4+ and CD8+ T cell responses in PBMC. (B) Representative ICS staining of CD8+ cells in Group 1 after the 2nd SHIV VLPs boost. Flow dot plots illustrate individual cytokine with no stimulation, HIV Env (N and C) peptides or with SIV Gag peptides pool stimulation. A positive control stimulation with PMA/Ionomycin was included in each assay. [file Image_1.tif]

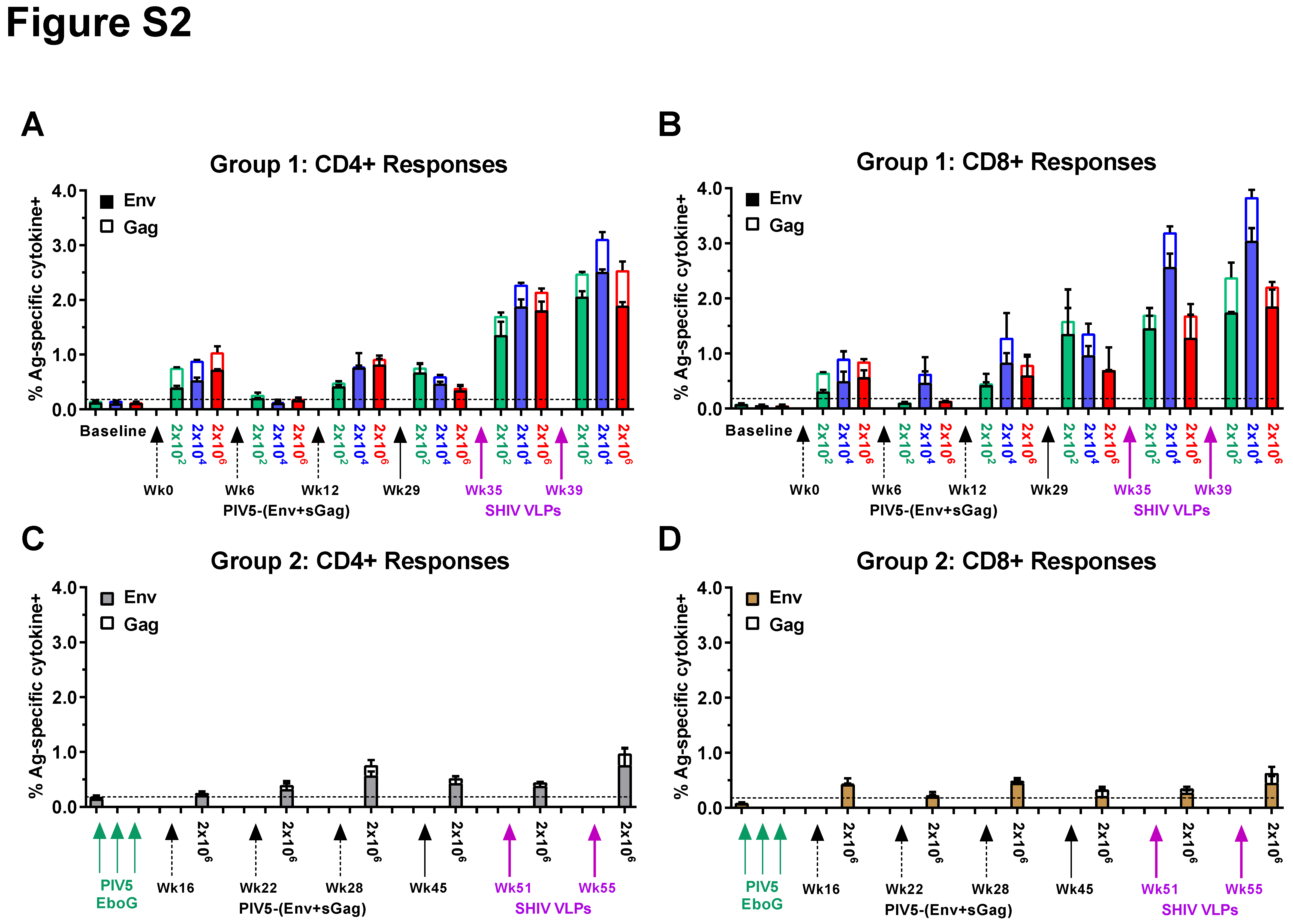

Supplement: Supplementary Figure 2 — Induction of antigen-specific cytokine producing CD4+ and CD8+ cells by intranasal administration of different PIV5 doses. (A, B) Group 1 of CD4+ or CD8+ T cells secreting cytokines specific for the sum of Env and Gag after administration with three different PIV5 doses of 2×10^2^ PFU (green), 2×10^4^ PFU (blue) and 2×10^6^ PFU (red), respectively. (C, D) Group 2 of CD4+ or CD8+ T cells secreting cytokines specific for the sum of Env and Gag after administration with a single PIV5 dose of 2×10^6^ PFU (grey or earth yellow). [file Image_2.tif]

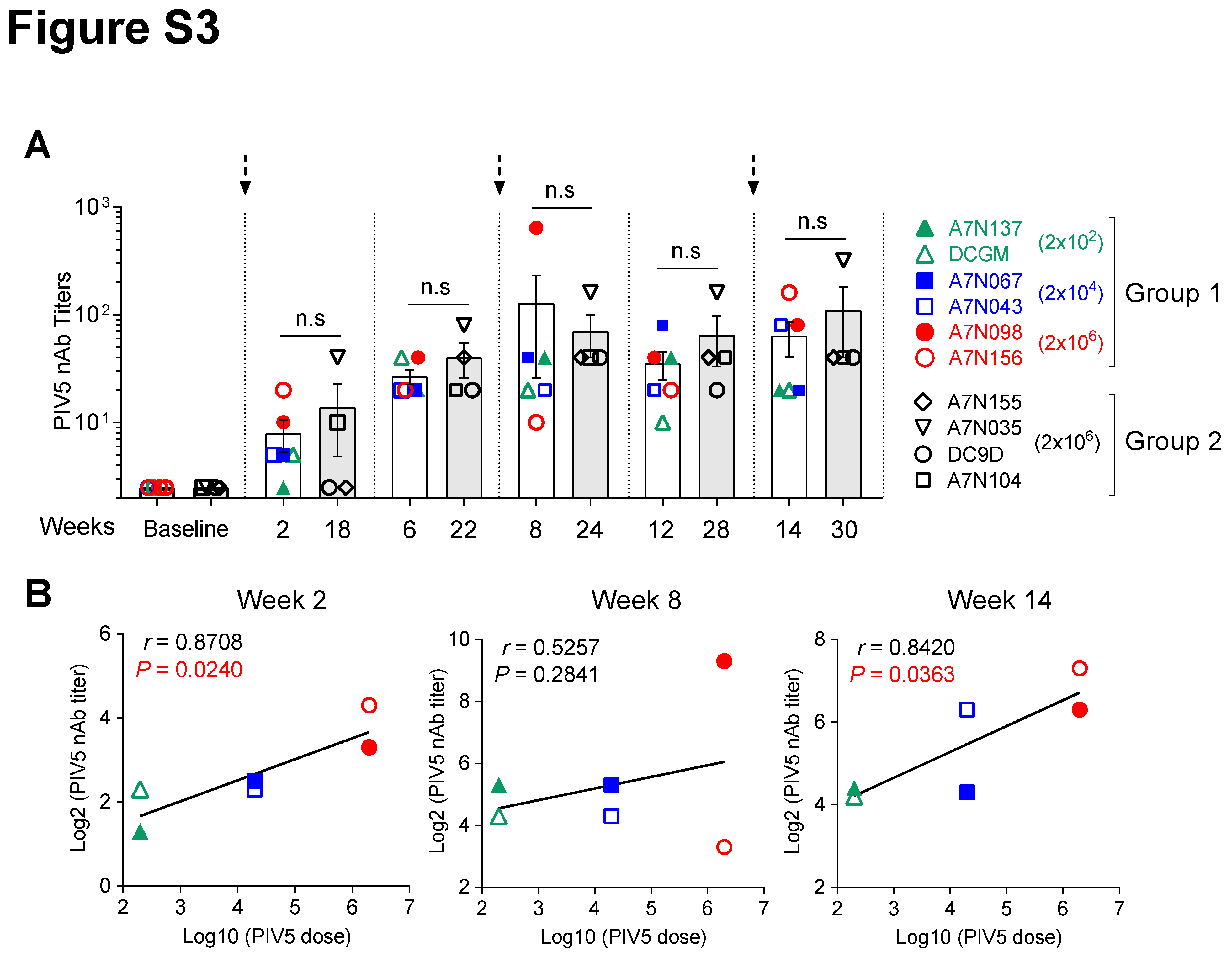

Supplement: Supplementary Figure 3 — (A) Comparison of titers of anti-PIV5 neutralizing antibodies between Group 1 and Group 2 at the indicated time points receiving the same immunization, respectively. (B) Correlation of anti-PIV5 titers relative to PIV5 doses at weeks 2, 8 and 14. The correlation coefficients (r) and P values were derived from Spearman rank analysis. Significant P values are in red font. [file Image_3.tif]
